# Hypoxia Changes Energy Metabolism and Growth Rate in Non-Small Cell Lung Cancer Cells

**DOI:** 10.3390/cancers15092472

**Published:** 2023-04-26

**Authors:** Hasan Nisar, Paulina Mercedes Sanchidrián González, Melanie Brauny, Frederik M. Labonté, Claudia Schmitz, Marie Denise Roggan, Bikash Konda, Christine E. Hellweg

**Affiliations:** 1 Department of Radiation Biology, Institute of Aerospace Medicine, German Aerospace Center (DLR), 51147 Cologne, Germanyfrederik.labonte@dlr.de (F.M.L.);; 2 Department of Medical Sciences, Pakistan Institute of Engineering and Applied Sciences (PIEAS), Nilore, Islamabad 44000, Pakistan; 3 Interfaculty Institute of Microbiology and Infection Medicine, Faculty of Science/Faculty of Medicine, University of Tübingen, 72074 Tübingen, Germany; 4 Department of Biology, Faculty of Mathematics and Natural Sciences, University of Cologne, 50923 Cologne, Germany; 5 Deutsches Zentrum für Neurodegenerative Erkrankungen (DZNE), 53127 Bonn, Germany

**Keywords:** lung cancer, non-small cell lung cancer cell lines, hypoxia, proliferation, energy metabolism, cell cycle progression, glucose, lactate, p53, cell migration

## Abstract

**Simple Summary:**

Non-small cell lung carcinoma (NSCLC), similarly to most other solid malignancies, frequently exhibits lack of oxygen (hypoxia), and this has been implicated as a potential cause of treatment resistance. A better understanding of NSCLC behavior under hypoxia in the context of its cellular energetics may lead to developing more effective treatment strategies. In this study, the effects of prolonged hypoxia on the energy metabolism and cellular proliferation of two different NSCLC cell lines were investigated. Glucose consumption and lactate production by cells indicated energy metabolism changes under hypoxia. Cellular proliferation decreased under hypoxia, as indicated by slower cell growth kinetics and cell cycle phase distribution over time. Additionally, RNA sequencing revealed differential expression of genes involved in cell migration under hypoxia.

**Abstract:**

Hypoxia occurs in 80% of non-small cell lung carcinoma (NSCLC) cases, leading to treatment resistance. Hypoxia’s effects on NSCLC energetics are not well-characterized. We evaluated changes in glucose uptake and lactate production in two NSCLC cell lines under hypoxia in conjunction with growth rate and cell cycle phase distribution. The cell lines A549 (p53 wt) and H358 (p53 null) were incubated under hypoxia (0.1% and 1% O_2_) or normoxia (20% O_2_). Glucose and lactate concentrations in supernatants were measured using luminescence assays. Growth kinetics were followed over seven days. Cell nuclei were stained with DAPI and nuclear DNA content was determined by flow cytometry to determine cell cycle phase. Gene expression under hypoxia was determined by RNA sequencing. Glucose uptake and lactate production under hypoxia were greater than under normoxia. They were also significantly greater in A549 compared to H358 cells. Faster energy metabolism in A549 cells was associated with a higher growth rate in comparison to H358 cells under both normoxia and hypoxia. In both cell lines, hypoxia significantly slowed down the growth rate compared to proliferation under normoxic conditions. Hypoxia led to redistribution of cells in the different cycle phases: cells in G1 increased and the G2 population decreased. Glucose uptake and lactate production increase under hypoxia in NSCLC cells indicated greater shunting of glucose into glycolysis rather than into oxidative phosphorylation compared to normoxia, making adenosine triphosphate (ATP) production less efficient. This may explain the redistribution of hypoxic cells in the G1 cell cycle phase and the time increase for cell doubling. Energy metabolism changes were more prominent in faster-growing A549 cells compared to slower-growing H358 cells, indicating possible roles for the p53 status and inherent growth rate of different cancer cells. In both cell lines, genes associated with cell motility, locomotion and migration were upregulated under chronic hypoxia, indicating a strong stimulus to escape hypoxic conditions.

## 1. Introduction

Imperfect angiogenesis during growth of malignant tumors results in hypoxia within the tumor parenchyma. This may be chronic (diffusion-limited), due to increasing distances between the proliferating tumor cells and the feeding vessel, or it may be acute (perfusion-limited), due to the poor architecture of the feeding vessel periodically resulting in its collapse [1,2]. The threshold of oxygen partial pressure for defining tumor hypoxia is the cut-off where cellular adaptation to reduced availability of oxygen may lead to treatment resistance. This is generally agreed upon in the literature as 10 mm of Hg (~1%), although cell type and environmental conditions play a role as well [3,4].

Cellular hypoxia in solid tumors results in wide-scale metabolic changes, which include changes in cellular energetics primarily mediated by the hypoxia-inducible factor 1 (HIF-1) pathway [5]. Hypoxia shifts cells toward glycolysis. However, tumor cells receiving adequate oxygen may still rely more on glycolysis rather than oxidative phosphorylation for their energy needs, a phenomenon referred to as the Warburg effect. Glycolysis produces 18 times less adenosine triphosphate (ATP) per glucose molecule in comparison to oxidative phosphorylation. Additionally, in proliferating tumor cells, glucose is needed as a source for the synthesis of DNA building blocks, such as ribose 5-phosphate and nucleotides. As a result, there is tremendous pressure on tumor cells to ensure adequate glucose supply. To meet this increased demand, glucose transporters, such as GLUT-1 (solute carrier family 2 member 1 (SLC2A1)) and GLUT-3 (solute carrier family 2 member 3 (SLC2A3)), are upregulated, along with several key glycolytic enzymes, such as hexokinase 1, 2 and 3; aldolase A and C; glyceraldehyde 3-phosphate dehydrogenase (GPDH); and lactate dehydrogenase (LDH) [5,6,7]. In this way, cellular hypoxia plays an important role in tumor cell proliferation, survival and, thus, treatment resistance in conjunction with an enhanced glycolysis-based energy metabolism. Increased glycolysis, along with diminished oxidative phosphorylation, under hypoxia results in greater lactate production. To prevent cytosolic acidification, lactate is excreted from the cell through monocarboxylate transporters, thereby decreasing extracellular pH [5]. The lactate-containing and acidic extracellular environment may promote tumor growth and angiogenesis, immune evasion and therapy resistance [8,9,10].

Changes in energy metabolism are also accompanied by alterations in cellular proliferation. These effects are largely dependent on cell type, as well as the severity and duration of hypoxia. Hypoxia has been demonstrated to inhibit proliferation of several cell types and a wide variety of cancer cell lines. This is mainly brought about by cell cycle arrest at the G1/S and G2/M junctions, and a variety of mechanisms are reported in the literature to explain how this happens [11,12]. One such mechanism is the increase in expression of the cyclin dependent kinase (CDK) inhibitors (CDKIs) p21 and p27 following p53 stabilization [12].

In 2022, lung cancer accounted for 25% of cancer-related deaths, and more than 80% of diagnosed cases were non-small cell lung carcinoma (NSCLC) [13]. NSCLC, being rapidly dividing, exhibits markedly increased glucose uptake in 18F-fluorodeoxyglucose (FDG) positron emission tomography (PET). PET studies utilizing hypoxia-specific radiotracers have shown that hypoxia is detectable in up to 80% of NSCLC patients belonging to all clinical stages [14,15]. GLUT1 expression is also increased in excised tumor tissue upon immunofluorescence staining [16]. Currently, the effects of cellular hypoxia on energy metabolism, cellular proliferation and cell cycle phase distribution in NSCLC and their interconnections are not well-characterized in the literature.

This study aimed to quantitatively evaluate changes in glucose uptake and lactate production in p53 wt (A549) and p53 null (H358) NSCLC cell lines [17] under chronic hypoxia (≤1% O_2_) in conjunction with changes in growth rate, cell cycle phase distribution and gene expression. This study offers basic insights into NSCLC cellular energetics and proliferation under prolonged moderate (1% O_2_) and severe (0.1% O_2_) hypoxia and considers the possible impact of p53 status on cell cycle and cell proliferation based on the selection of the two cell lines.

## 2. Materials and Methods

### 2.1. Cell Lines and Cultivation

Two human NSCLC cell lines, A549 and H358, were obtained from LGC Genomics (Berlin, Germany; the European Partner of American Tissue Culture Collection (ATCC), Manassas, VA, USA). The cells were regularly tested for mycoplasma contamination by polymerase chain reaction of supernatants at the Leibniz-Institut DSMZ—Deutsche Sammlung von Mikroorganismen und Zellkulturen GmbH (Braunschweig, Germany) and were mycoplasma-free. They were routinely cultured in 80 cm^2^ cell culture flasks (Labsolute, Th. Geyer GmbH, Renningen, Germany) with Alpha-Minimally Essential Medium (α-MEM; PAN Biotech, Aidenbach, Germany) containing 2% (*v*/*v*) sterile glucose solution (0.94 mol/L) and 10% (*v*/*v*) dialyzed fetal bovine serum (FBS; PAN Biotech) (complete α-MEM), as well as 1% (*v*/*v*) penicillin (10,000 U/mL)/streptomycin (10 mg/mL) (PAN Biotech), 1% (*v*/*v*) neomycin/bacitracin (1 mL bacitracin (2500 U/mL) mixed in 50 mL neomycin sulfate (10 mg/mL)) (Biochrom AG, Berlin, Germany) and 1% (*v*/*v*) amphotericin (250 µg/mL) (PAN Biotech). They were incubated at 37 °C and saturated humidity under normoxia (20% O_2_) in a CO_2_ incubator (Heraeus HERAcell 150, Thermo Fisher Scientific, Braunschweig, Germany) or under hypoxia (0.1% O_2_ and 1% O_2_). Hypoxia was maintained by incubating and handling cells in an InvivO2 400 hypoxia workstation (Baker Ruskinn, South Wales, UK) flushed with 5% CO_2_, 1% or 0.1% O_2_ and 94% or 94.9% N_2_, respectively. The total time cells remained in culture under normoxia or hypoxia depended on the type of experiment: 72 h for metabolic assays (Section 2.2), 7 days for determination of cell proliferation (Section 2.3) or 48 h pre-incubation plus 24 h follow-up for analysis of cell cycle distribution (Section 2.4).

### 2.2. Quantification of Glucose Consumption and Lactate Production

A549 and H358 cells were seeded in Petri dishes (∅ 6 cm; Labsolute, Th. Geyer GmbH, Germany), in 5 mL each of complete α-MEM, at a density of 5000 cells/cm^2^ and 20,000 cells/cm^2^, respectively. Cells were incubated for up to 3 days under either hypoxia (0.1% O_2_ and 1% O_2_) or normoxia (20% O_2_), as described in Section 2.1. During this 3-day time period, cell culture supernatants were collected at 24 h time intervals from three Petri dishes per day and cells were trypsinized for cell counts as described below. The supernatants and an aliquot of the culture medium (for baseline glucose and lactate concentration without cells) were stored at −80 °C until analysis. Energy metabolism of the cell lines was assessed in terms of glucose consumption and lactate production by measuring glucose and lactate concentrations in the supernatants with the Promega Glucose-Glo^TM^ and Lactate-Glo^TM^ luminescence assays (Promega GmbH, Walldorf, Germany) [18,19]. Supernatants and culture medium were thawed and diluted with phosphate buffered saline (PBS) to a final concentration of 1:500 for the glucose assay and 1:100 for the lactate assay. The reagents in the assay kits were mixed according to the manufacturer’s protocols and the diluted sample supernatants were added to the reagent mixture (1:1; 50 µL each) in a Corning round-bottom 96-well plate (Stemcell Technologies, Vancouver, BC, Canada). Additionally, known concentrations of glucose (10 to 50 µmol/L) and lactate (10 to 200 µmol/L) were loaded into the 96-well plate to obtain a standard curve. PBS was used as a glucose-free negative control. Luminescence from the samples was measured using the Infinite M200Pro multimode microplate reader (Tecan, Groeding, Austria) after shaking the 96-well plate for 60 s and then incubating it for 1 h at 25 °C. Based on the standard curve, glucose and lactate concentrations in the supernatants and culture medium were derived from the luminescence measurements of the remaining glucose in the medium. The amount of glucose taken up by the cell layers and the amount of lactate produced by the cell layers were calculated by multiplying the respective concentrations by the α-MEM volume used in the Petri dishes. Glucose uptake into the cells was calculated by subtracting the measured amount of glucose in the supernatants from the baseline amount in the culture medium. Extracellular lactate secretion was calculated by subtracting the baseline amount of lactate in the culture medium from the amount measured in the collected supernatant.

To determine the number of viable cells in each sample, Petri dishes for each time point were washed with PBS at the appropriate time, cells were trypsinized (0.05% trypsin, 0.02% EDTA; PAN Biotech) and the cell suspension was stained with Trypan Blue (1:1). The Trypan Blue-negative viable cells were counted using a LUNA^TM^ automated cell counter (Logos Biosystems, Gyeonggi-do, South Korea). The number of viable cells was used to normalize glucose and lactate concentrations in each supernatant to account for varying cell proliferation rates at different oxygen concentrations.

### 2.3. Determination of Cell Proliferation

A549 and H358 cells were seeded in 3 mL each of complete α-MEM in Petri dishes (∅ 3 cm; Labsolute, Renningen, Germany) at a density of 5000 cells/cm^2^ and 20,000 cells/cm^2^, respectively. Cells were incubated at the different oxygen concentrations as described in Section 2.1 in three Petri dishes per day. Viable cells were counted daily using LUNA^TM^ over a seven-day period. Culture medium was replaced on the fourth day of incubation while maintaining the respective oxygen concentrations of the samples. The number of viable cells per cm^2^ growth area was calculated to generate growth curves and derive the cell doubling times. Cell doubling times (t_d_) were calculated for each cell line and oxygen concentration using the formula suggested by Korzynska et al. [20].

### 2.4. Analysis of Cell Cycle Distribution

In separate experiments, the two cell lines were cultured in Petri dishes (∅ 6 cm) at the same seeding densities as mentioned above and then pre-incubated for at least 48 h at 1% and 20% O_2_, as described in Section 2.1. Following pre-incubation, over the next 24 h, cells were detached with 1 mL Trypsin/EDTA solution and fixed in 3.5% formaldehyde at defined time points (2 h, 6 h, 12 h, 18 h and 24 h after incubation). Then, 30 min after fixation, the cells were washed in PBS and the cell nuclei of the two cell lines were stained with freshly prepared 4′,6-diamidino-2-phenylindole (DAPI) solution (500 ng/mL) and Triton X (3 µg/mL) in PBS and kept in the dark for 30 min at room temperature. The nuclear DNA content of the two cell lines was measured using flow cytometry (Cytoflex S, Beckman Coulter, Indianapolis, IN, USA) to determine the cell cycle phase distribution under hypoxia compared to normoxia. A violet laser (405 nm) was used as excitation source. Blue fluorescence was measured in the fluorescence channel PB450 of the flow cytometer. After forward- and side-scatter and PB450 width/area gating, the blue fluorescence histogram of the single cells was analyzed in FloJo software (v. 10) with automated cell cycle analysis using the Dean-Jett-Fox cell cycle mathematical model [21].

### 2.5. Gene Expression Analysis

The global transcription profile during exposure to hypoxia (1% O_2_) was analyzed by mRNA sequencing. A549 and H358 cells were seeded in 25 cm^2^ culture flasks (LABsolute, Th. Geyer GmbH, Germany) in 5 mL standard culture medium each from confluent 75 cm^2^ tissue culture flasks (Nunc^TM^) at a density of 5000 cells/cm^2^ and 20,000 cells/cm^2^, respectively. The cells were incubated under either normoxia or hypoxia for 52 h (see Section 2.1). For harvesting after 52 h of incubation, the medium was completely removed and cells were lysed using RLT buffer (Qiagen, Hong Kong, China) with β-mercaptoethanol (1:100, Sigma Aldrich, St. Louis, MI, USA). The homogenized lysate was stored at −80 °C until RNA isolation with the RNeasy Mini Kit on the same day for all samples. RNA concentration and integrity were determined by means of the RNA 6000 Nano Assay in a Bioanalyzer (Agilent Technologies, Böblingen, Germany). RNA integrity numbers (RIN) of all samples were above 9.0. At least 3 µg of total RNA per sample (four biological repeats per condition) was sent on dry ice to GENEWIZ (Leipzig, Germany) for mRNA sequencing in the same run after Poly(A) selection using the Illumina NovaSeq6000 platform (configuration: 2 × 150 bp, 350 M read pairs) and bioinformatics analysis, including trimming, mapping, batch correction and differential gene expression, following the principles described in [22]. Significantly differentially expressed genes were identified using the Wald test for calculation of *p*-values and the Benjamini–Hochberg test for calculation of adjusted *p*-values (*p*adj). Genes with a *p*adj < 0.05 and absolute log2 fold change > 1 were considered as differentially expressed genes for each group comparison. They were then were clustered according to their gene ontology (GO) and the enrichment of GO terms was tested using the Fisher exact test (GeneSCF v1.1-p2).

### 2.6. Statistical Analysis

Three independent biological experiments with three technical replicates for each experimental condition were conducted for the experiments described in Section 2.2, Section 2.3 and Section 2.4. Arithmetical means, standard deviations and standard errors of means (SEs) were calculated using Excel (2019 MSO, Microsoft, Albuquerque, NM, USA). Graphs were plotted, and tests of significance were performed using GraphPad Prism 9 (Dotmatics, Boston, MA, USA). Significance was tested using one-way ANOVA to evaluate cell metabolism studies and multiple two-way unpaired *t*-tests were used to evaluate cell cycle distribution data and doubling times. Growth curves were plotted using Sigma Plot 15 (Systat Software Inc., Palo Alto, CA, USA). For the RNA sequencing, four independent biological experiments were conducted and a batch analysis of the results was performed. A batch effect was observed for H358 cells; therefore, two samples had to be excluded from further analysis.

## 3. Results

### 3.1. Glucose Uptake Increases under Hypoxia in NSCLC Cell Lines

**Figure 1:**
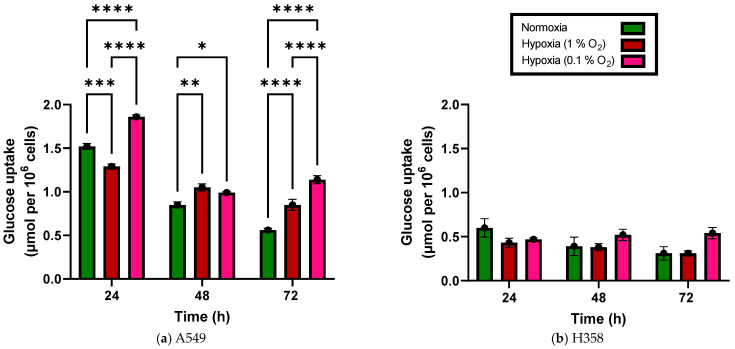
Glucose uptake of A549 cells (**a**) and H358 cells (**b**) grown with 20%, 1% and 0.1% oxygen 24 h, 48 h and 72 h after seeding (additive values). *: *p* < 0.05; **: *p* < 0.01; ***: *p* < 0.001; ****: *p* < 0.0001; *n* = 3. Error bars represent SE.

In the case of the A549 cells, glucose uptake per 10^6^ cells 48 and 72 h after the start of incubation under hypoxia (0.1% and 1% O_2_) was significantly greater than that under normoxia (Figure 1a). Even after 24 h, a significant increase in glucose uptake was observed under severe hypoxia (0.1% O_2_) but not when cells were incubated in the presence of 1% O_2_. H358 cells showed a similar trend under hypoxia compared to normoxia, particularly at 0.1% O_2_, but the increase in glucose uptake did not reach statistical significance (Figure 1b).

### 3.2. Lactate Secretion Increases under Hypoxia in NSCLC Cell Lines

**Figure 2:**
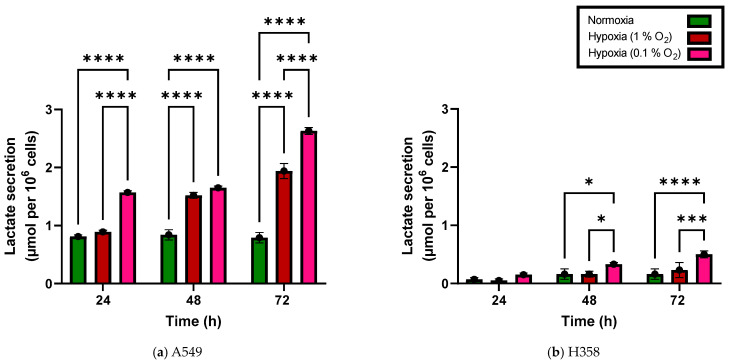
Lactate secretion of A549 cells (**a**) and H358 cells (**b**) grown with 20%, 1% and 0.1% oxygen 24 h, 48 h and 72 h after seeding (additive values). *: *p* < 0.05, ***: *p* < 0.001, ****: *p* < 0.0001; *n* = 3. Error bars represent SE.

Lactate production and secretion per 10^6^ cells under hypoxia was also found to be greater than that under normoxia, indicating that excessive glucose uptake was not accompanied by a matching increase in oxidative phosphorylation (Figure 2). The increase was significant in A549 cells incubated for 24, 48 and 72 h at either 0.1% or 1% O_2_ relative to normoxia. H358 cells showed a similar trend after incubation for 24, 48 and 72 h at both 0.1% and 1% O_2_ and this increase in lactate production under hypoxia reached statistical significance in H358 cells after incubation periods of 48 and 72 h. At any incubation time point, glucose consumption and lactate production—normalized to the cell number—were significantly greater in A549 cells compared to H358 cells.

### 3.3. NSCLC Cellular Proliferation Rate Decreases under Hypoxia

**Figure 3:**
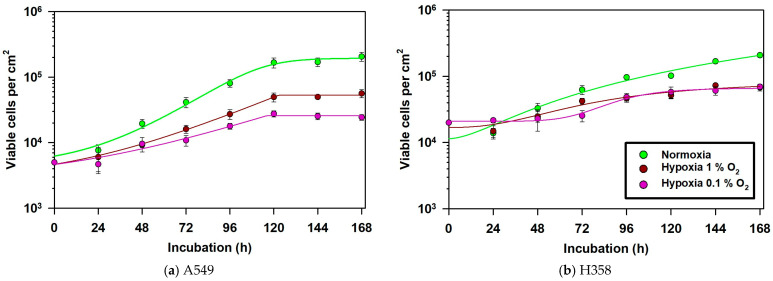
Growth kinetics of A549 cells (**a**) and H358 cells (**b**) growing with 20%, 1% and 0.1% O_2_ over 168 h (7 days). Culture medium was refreshed on day 4 of cell culturing. Cells were counted with an automated cell counter after staining them with Trypan Blue to identify living cells. *n* = 3. Error bars represent SE.

Cellular energetics in A549 and H358 cells were correlated with changes in their growth rate under hypoxia. A higher basal energy metabolic turnover in A549 cells in comparison to H358 cells was also associated with their relatively higher growth rate, which resulted in earlier achievement of the stationary phase by normoxic A549 cells compared to normoxic H358 cells (Figure 3). The smaller doubling time (t_d_) of 23.5 h in normoxic A549 cells compared to 33.3 h in H358 cells indicated the higher growth rate of A549 cells during the exponential growth phase (Table 1). Incubation at 1% and 0.1% O_2_ increased t_d_ in A549 cells by 32% and 128% in comparison to normoxic controls, indicating slower growth under hypoxia. Similarly, H358 cells showed increases in t_d_ of 57% and 106% in comparison to normoxic controls when incubated at 1% and 0.1% O_2_, respectively. Hypoxia significantly slowed the growth rate in both cell lines (Table 2) and it also significantly decreased the maximal cell density reached in the saturation phase of the growth curve (Figure 3 and Table 2, 144 h and 168 h values).

### 3.4. NSCLC Cell Lines Show Cell Cycle Phase Redistribution toward G1 under Hypoxia

**Figure 4:**
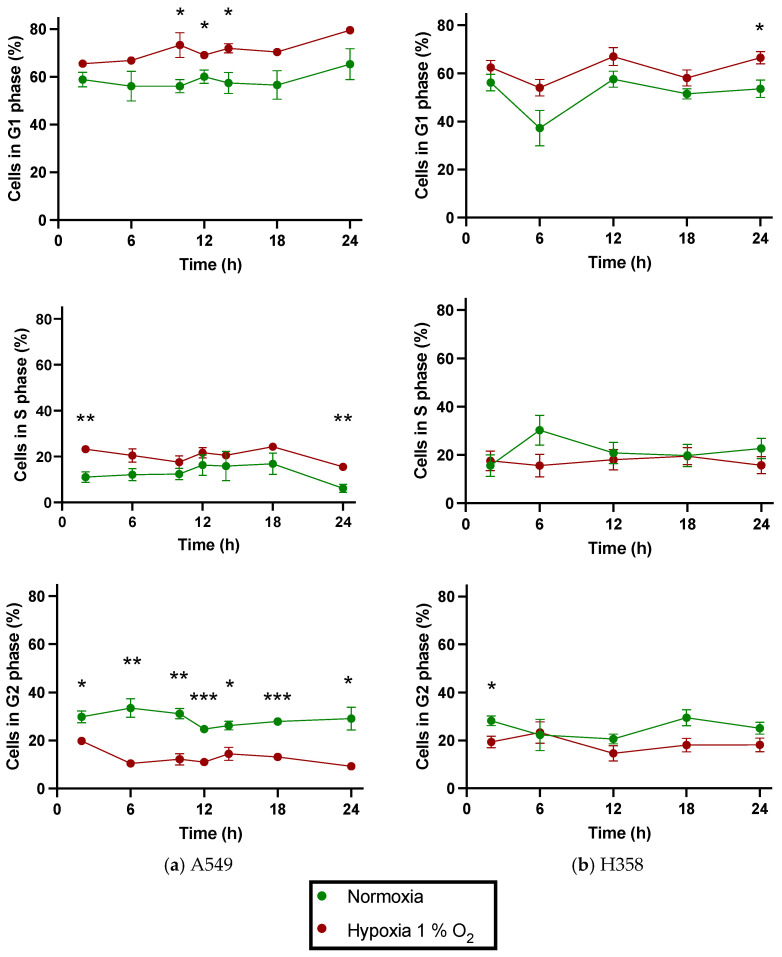
Distribution of A549 cells (**a**) and H358 cells (**b**) in G1 (top), S (middle) and G2 (bottom) phases of the cell cycle over 24 h following pre-incubation of 48 h with 20% or 1% O_2_. A period of 48 h was selected for the pre-incubation period to evaluate the effect of chronic hypoxia and to allow cells to overcome the lag phase of the growth curve. *: *p* < 0.05, **: *p* < 0.01, ***: *p* < 0.001; *n* = 3. Error bars represent SE.

Hypoxia-induced changes in the growth rates of A549 and H358 cells were accompanied by greater cell cycle phase redistribution of hypoxic cells compared to normoxic cells. Cell populations in different cell cycle phases were followed periodically over 24 h after a pre-incubation period of 48 h. During this total period spanning over 72 h, normoxic cells remained in a normoxic environment while hypoxic cells were maintained at 1% O_2_. Both A549 and H358 cells growing at 1% O_2_ showed an increased proportion of cells in G1 compared to normoxic controls when evaluated periodically over the next 24 h. Similarly, the proportion of cells in G2 was lower under hypoxia compared to normoxia (Figure 4).

Under normoxia, there was no significant difference in the distribution of the cell populations in the G1 and G2 phases of the cell cycle when comparing A549 and H358 cells; however, under hypoxia, a significantly greater proportion of A549 cells were found in G1—and, consequently, a smaller proportion of cells in G2—when compared with H358 cells at several time points (Table 3).

**Figure 5:**
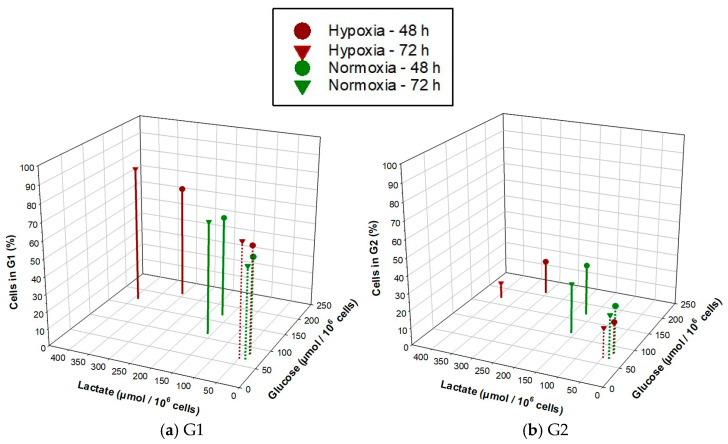
Relationship between glucose uptake and lactate secretion and the percentage of cells in the G1 (**a**) and G2 (**b**) phases of the cell cycle for A549 (solid lines) and H358 (dotted lines) cells growing under normoxia and hypoxia (1% O_2_) for 48 and 72 h.

To understand the relationship between glucose uptake and lactate secretion and the distribution of cells in the cell cycle for the two NSCLC cell lines under normoxia and hypoxia, a three-dimensional plot was created (Figure 5). Under hypoxia, changes in energy metabolism were greater for A549 cells in comparison to H358 cells, while G1 populations tended to remain higher and G2 populations tended to remain lower under hypoxia for both cell lines in comparison to normoxia.

### 3.5. NSCLC Cell Lines Show Distinct Gene Expression Changes under Hypoxia

**Figure 6:**
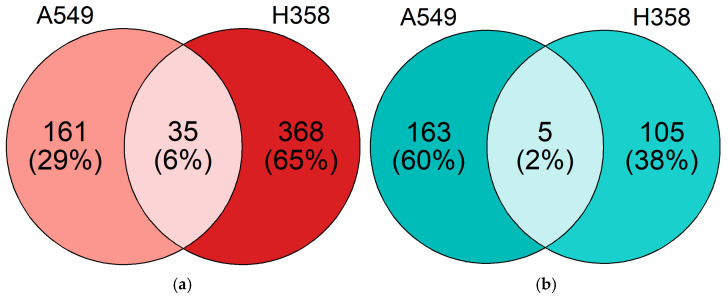
Venn diagram of gene expression changes in A549 and H358 cells growing under normoxia or hypoxia (1% O_2_) for 52 h. Gene expression was analyzed using RNA sequencing (A549: *n* = 4; H358: *n* = 2). The numbers of significantly differentially expressed genes (DEGs) based on a log2 fold change above 1 in A549 cells only, in H358 cells only and in both cell lines are indicated. (**a**) Genes with expression upregulated under hypoxia. (**b**) Genes with expression downregulated under hypoxia.

**Figure 7:**
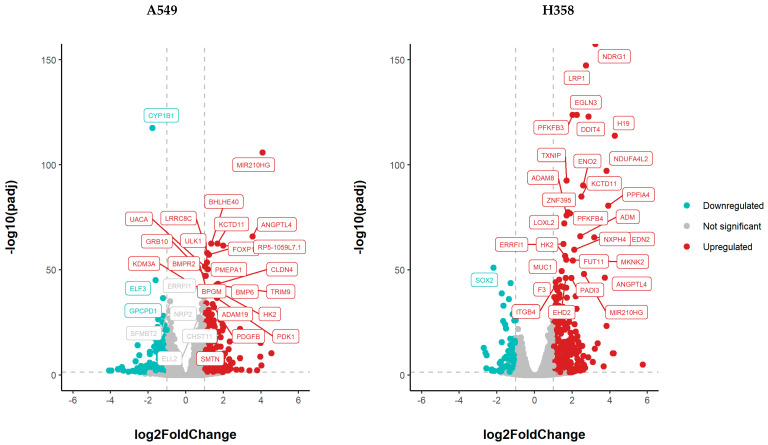
Gene expression changes in A549 and H358 cells growing under hypoxia (1% O_2_) for 52 h in comparison to normoxic controls. Gene expression was analyzed using RNA sequencing (A549: *n* = 4; H358: *n* = 2). In these volcano plots, the log_10_ of the adjusted *p* value (log_10_ (*p*adj)) is plotted against the log_2_ fold change in the expression of each gene. Some of the most significantly upregulated genes (red dots) are annotated and a few downregulated genes (blue-green dots) are also shown. The full list of significant DEGs is available in the Supplementary Materials.

Through RNA sequencing, changes in gene expression under hypoxia compared to normoxic incubation were quantified after 52 h of hypoxic incubation (1% O_2_) of both cell lines. A total of 40 genes were differentially regulated under hypoxia in both NSCLC cell lines; 324 genes were regulated exclusively in A549 cells under hypoxia, while 473 genes were exclusively regulated in H358 cells under hypoxia (Figure 6 and Figure 7).

To determine whether genes involved in cell cycle regulation were differentially expressed under hypoxia, the significant DEGs for both cell lines were evaluated using a list of 129 established cell cycle genes available in the KEGG database [23]. Hypoxic A549 cells showed no differential regulation of cell cycle genes compared to normoxic controls, while the slower-dividing hypoxic H358 cells showed differential downregulation of CDKN1C (p57) with a log_2_ fold change of −1.42 (*p*adj = 1.15 × 10^−3^) in comparison to normoxic controls.

Significant DEGs for both cell lines under hypoxia were also evaluated using a list of 144 established genes involved in regulation of glycolysis and oxidative phosphorylation available in the KEGG database [23]. In hypoxic A549 cells, bisphosphoglycerate mutase (BPGM) was upregulated with a log_2_ fold change of 1.09 (*p*adj = 8.89 × 10^−35^). In hypoxic H358 cells, enolase 2 (ENO2, log_2_ fold change of 2.58, *p*adj = 6.88 × 10^−91^) and NADH dehydrogenase (ubiquinone) 1 alpha subcomplex subunit 4-like 2 (NDUFA4L2, log_2_ fold change of 3.82, *p*adj = 7.76 × 10^−98^) were upregulated.

**Figure 8:**
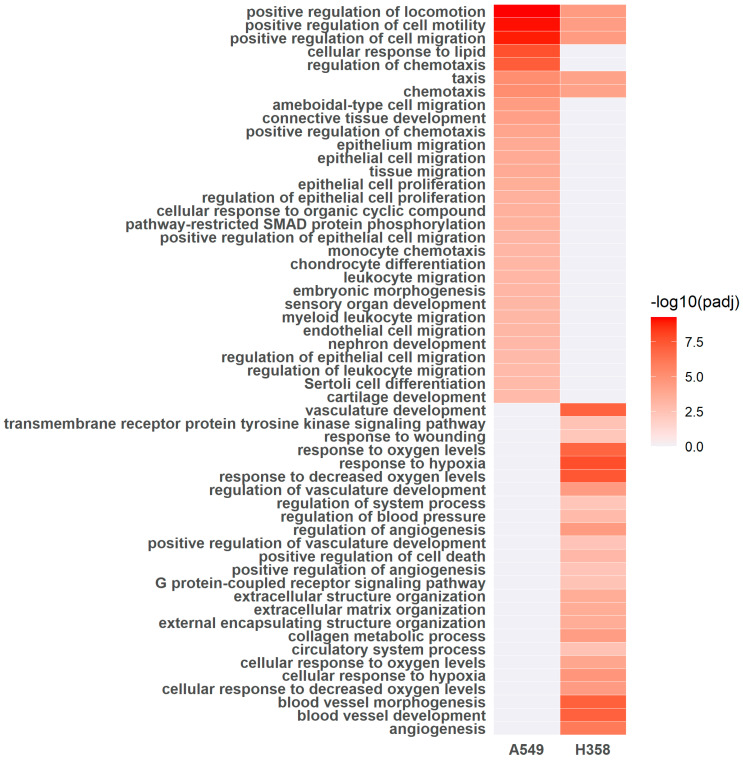
Gene ontology terms for regulated genes in A549 and H358 cells growing under hypoxia (1% O_2_) for 52 h in comparison to normoxic controls. Gene expression was analyzed using RNA sequencing (A549: *n* = 4; H358: *n* = 2). Gene ontology (GO) terms that were significantly enriched with an adjusted *p* value lower than 0.05 in the differentially expressed gene sets are listed (up to 60 terms with five overlaps for both cell lines). The full list of enriched GO terms is available in the Supplementary Materials.

Furthermore, gene ontology (GO) terms for regulated genes in A549 and H358 cells growing under hypoxia (1% O_2_) were determined in comparison to normoxic controls. The top 30 GO terms are shown in Figure 8. GO terms for locomotion, motility, migration and (chemo-)taxis overlapped for both cell lines. These GO terms are closely related. Five genes upregulated under hypoxia in both cell lines served to enrich all the aforementioned overlapping GO terms. These genes were: platelet derived growth factor β (PDGFB, log_2_ fold change in A549 of 1.50, *p*adj = 6.35 × 10^−33^, and log_2_ fold change in H358 of 2.19, *p*adj = 2.68 × 10^−21^); tissue factor (F3, log_2_ fold change in A549 of 1.71, *p*adj = 4.28 × 10^−6^, and log_2_ fold change in H358 of 1.11, *p*adj = 6.71 × 10^−45^); serine protease inhibitor clade E member 1 (SERPINE 1, log_2_ fold change in A549 of 1.53, *p*adj = 1.65 × 10^−23^, and log_2_ fold change in H358 of 2.11, *p*adj = 1.58 × 10^−12^); semaphorin 4B (SEMA4B, log_2_ fold change in A549 of 1.14, *p*adj = 1.85 × 10^−13^, and log_2_ fold change in H358 of 1.20, *p*adj = 1.72 × 10^−41^); and vascular endothelial growth factor α (VEGFA). The non-overlapping GO terms for hypoxic A549 cells mostly referred to cell migration, while in hypoxic H358 cells, a trend toward a hypoxia response and angiogenesis was apparent.

## 4. Discussion

This study compares the effect of chronic hypoxia (1% and 0.1% O_2_) on energy metabolism and proliferation rate in the rapidly dividing (t_d_ 23.5 h) p53 wt A549 and relatively slower-growing p53 null H358 (t_d_ 33.3 h) NSCLC cell lines. In both cell lines, a KRAS missense mutation is present (H358: KRAS-G12C, A549: KRAS-G12S), as reported in the supplier (ATCC) product sheet, and they are histologically characterized as adenocarcinoma [17]. Additionally, this study compared cell cycle phase distribution and gene expression in the two cell lines under chronic hypoxia (1% O_2_).

The metabolic plasticity of cancer cells is understood in the literature to be a mechanism for tumor survival and proliferation [22,23]. Both cell lines tended to exhibit an increase in glucose uptake under incubation at 0.1% O_2_, but it was statistically significant only in A549 cells. The greater glucose uptake is indicative of greater shunting of glucose into the glycolytic pathway under hypoxia compared to normoxia. Furthermore, lactate production increased statistically significantly in both cell lines under hypoxia, indicating that increased glycolysis is not coupled to an equivalent increase in oxidative phosphorylation, which explains why we observed excess pyruvate conversion into lactate. This was not unexpected under hypoxia; cancer cells in particular, and rapidly proliferating cells in general, tend to rely more on glycolysis than oxidative phosphorylation for their energy and metabolic needs [24].

While A549 cells were quick to show a significant increase in glucose uptake and lactate production at both 1% and 0.1% O_2_, the H358 cells were much slower to respond to hypoxia, demonstrating a statistically significant increase in glycolysis relative to normoxia only in terms of lactate production, and only when incubated at 0.1% O_2_ and not at 1% O_2_. Whether this was due to an inherently slower proliferation rate in the H358 cells, apoptosis or their p53 null status remains to be understood. Hypoxia can induce p53 secondary to an increase in the ADP/ATP intracellular ratio [25,26], with the overall effect of p53 activation being the slowing down of cellular proliferation and maintenance of cellular energy metabolism [27,28,29]. In the mitochondria, p53 can augment oxidative phosphorylation through increased expression of synthesis of cytochrome c oxidase protein (SCO2), which is needed for proper assembly of cytochrome c oxidase [30,31]. After 52 h under hypoxia, SCO2 was not differently regulated in A549, but early transient upregulation cannot be excluded. Selective transient p53 activation in A549 but not H358 might, therefore, explain the higher glucose consumption in the former under hypoxia. Guo et al. observed activation of p53 in A549 cells starting within 4 h of hypoxic (1% O_2_) incubation [32]. In this study, after 52 h under hypoxia, distinct activation of the transcriptional action of p53 was not detected in the RNA Seq dataset, suggesting that p53 activation under hypoxia might be transient. The impact of the slower adaptation of energetics in H358 may indicate a weaker adaptive ability to survive and multiply under hypoxia. A lack of metabolic flexibility was recently also observed in head and neck cancer cells that lost p53 function [33].

In a quantitative proteomic approach, enzymes involved in glycolysis and the pentose phosphate pathway were upregulated in A549 compared to a non-tumoral bronchial cell line (BEAS-2B) [34]. This could be an explanation of the high glucose consumption and lactate production in A549. In our RNA Seq dataset, BPGM was upregulated in hypoxic A549 cells in comparison to their normoxic controls. BPGM converts 3-bisphosphoglycerate (BPG) to 2,3-BPG, which is then metabolized to 3-phosphoglycerate (3-PGA), an intermediate of the glycolytic pathway. Its upregulation is therefore consistent with an increase in glycolysis in hypoxic A549 cells.

H358 cells, on the other hand, showed upregulation of ENO2, which is responsible for converting 2-phosphoglycerate (2-PG) to phosphoenolpyruvate (PEP), a rate-limiting step in glycolysis. Its upregulation is therefore consistent with an increase in glycolysis in hypoxic H358 cells, as evidenced by increased lactate production under hypoxia compared to normoxia (Figure 2b). Additionally, hypoxic H358 cells also exhibited upregulation of NDUFA4L2 in our study. This is a component of the electron transport chain (ETC) complex I subunit and its upregulation under hypoxia mediates inhibition of oxidative phosphorylation [35], which may offer a mechanistic explanation for the decoupling of glycolysis and oxidative phosphorylation under hypoxia in H358 cells. As two experiments had to be excluded from the gene expression analysis due to a batch effect, the H358 gene expression changes should only be interpreted as trends.

Despite an increase in glycolysis, the cellular proliferation rate in hypoxic NSCLC cells decreased, as exemplified by the increase in their doubling times. Although this increase in t_d_ was greater for the metabolically slower-adapting H358 cells (57%) compared to A549 (32%) cells at 1% O_2_, this was not the case at 0.1% O_2_, where the metabolically faster-adapting A549 cells showed a 128% increase in t_d_ compared to 106% for H358. The greater slowing down of A549 cells compared to H358 cells at 0.1% O_2_ may be attributable to lower extracellular pH due to greater production of lactic acid in the former cell line, which is known to negatively impact cellular proliferation [36].

Cell cycle phase distributions in A549 and H358 cells under hypoxia (1% O_2_) were evaluated to search for a possible mechanism for the reduced cellular proliferation at reduced oxygen concentrations. The increase in the G1 cell population and decrease in the G2 cell population in both A549 and H358 cells when incubated at 1% O_2_ appears to be the likely explanation for the slowing down of their proliferation rate under chronic hypoxia. However, in H358 cells, the cell cycle phase redistribution trend under hypoxia did not show statistical significance at most of the time points used for cell cycle analysis. While both cell lines showed no significant difference in cell cycle phase distribution when compared to each other under normoxia, the hypoxic A549 cells showed significantly higher G1 and lower G2 populations compared to hypoxic H358 cells. Additionally, A549 cells seemed to additionally exhibit S phase arrest under continuous hypoxia with 1% O_2_. These findings may be once again explained based on the difference in p53 status between the two cell lines. Under hypoxia, a functional TP53 gene translates into p53 protein, the upregulation of which causes an increase in p21 protein levels, which can be responsible for cell cycle arrest in the G1 phase [32,37]. Similarly, hypoxia can also lead to S phase cell cycle arrest through downregulation of cyclin A, which is a known target of p21 [38,39]. In contrast, hypoxia-induced cell cycle arrest in H358 cells must occur through p53-independent mechanisms, mainly at the G2/M checkpoint, resulting in less pronounced cell cycle alterations compared to A549 cells.

However, in our study, we could find no transcriptional basis for the observed redistribution of hypoxic NSCLC cells toward G1 and away from G2. Most probably, cell cycle genes were not found to be differentially regulated in hypoxic A549 and H358 cells compared to normoxic controls because the 52 h time point for RNA collection was too late to capture changes in cell cycle regulation at the transcriptional level. The slower-dividing hypoxic H358 cells, however, did show differential downregulation of CDKN1C (p57) in comparison to normoxic controls. CDKN1C is primarily a G1/S checkpoint inhibitor implicated in the inhibition of cyclin E-CDK2, cyclin D2-CDK4 and cyclin A-CDK2. Its downregulation may be a counter-homeostatic strategy to overcome G1 inhibition in H358 cells following prolonged hypoxia (52 h).

While gene ontology enrichment analysis (Figure 8) revealed no enriched GO terms directly pertaining to metabolism or cellular proliferation, the results were still exciting because it was seen that prolonged incubation (52 h) of A549 and H358 cells under hypoxia was associated with gene expression changes leading to enhanced cell motility and migration. Such modifications are increasingly being reported to result in enhanced metastatic potential and, hence, tumor aggressiveness and treatment resistance [40,41,42]. In fact, increased lactate production has been reported to be a direct cause of increased cell migration and invasion through the activation of proteases, including matric metalloproteases (MMPs), urokinase-type plasminogen activator and cathepsins, in response to low extracellular pH; additionally, lactate can stabilize HIF-1α, which in turn can promote cell migration [43]. The predominance of GO terms relating to motility and migration suggests that chronic hypoxia is a strong signal for A549 and H358 cells to move to regions with more favorable oxygen conditions.

Furthermore, we identified five differentially upregulated genes (PDGFB, F3, SERPINE1, SEMA4B and VEGFA) under hypoxia involved in enrichment of the five GO processes pertaining to cell motility and migration in both A549 and H358 cells. PDGFB is understood to be a major contributor in malignant transformations through the induction of the TGFβ pathway, resulting in increased extracellular matrix deposition and epithelial-mesenchymal transition (EMT) [44]. F3 has been reported to induce cell migration via p38 activation and can help tumor cells to escape immune surveillance through recruitment of myeloid-derived suppressor cells (MDSCs) into the tumor microenvironment [45]. SERPINE1 has been shown to enable tumor microenvironment remodeling and immune cell infiltration, particularly in gastric carcinoma [46,47]. SEMA4B is a transmembrane glycoprotein that has been reported to be overexpressed in lung adenocarcinoma, which has been correlated with poor prognosis [48]. VEGFA promotes cancer cell renewal, cell migration, invasion and metastasis through induction of SOX-2, which in turn activates SNAI2, an EMT transcription factor [49]. Its inhibition through the use of angiotensin converting enzyme II (ACE II) inhibitors has been shown to restrain cancer progression in NSCLC [50].

## 5. Conclusions

Prolonged hypoxia (> 48 h) results in changes in cellular energy metabolism, proliferation and cell migration in NSCLC cell lines, regardless of p53 status. Both p53 wt (A549) and p53 null (H358) NSCLC cell lines at 0.1% and 1% O_2_ showed adaptation of energy metabolism—an increase in glycolysis without an increase in oxidative phosphorylation—manifesting as an increase in lactate secretion. This was accompanied by a decline in proliferation rates for the two cell lines under hypoxia. However, differences in metabolic response to hypoxia in the context of cellular energetics did not translate into a noteworthy difference in the extent to which the doubling times of the two cell lines lengthened under hypoxia. The decline in cellular proliferation seemed to manifest due to redistribution of cells in the G1 phase and away from the G2 phase of the cell cycle. Global transcription studies comparing the two NSCLC cell lines after 52 h of hypoxia (1% O_2_) to their normoxic controls revealed minimal changes in the expression of cell cycle genes and those involved in energy metabolism, probably because of the late time point for RNA collection. On the other hand, the differentially expressed genes under hypoxia at this time point showed enrichment of GO processes pertaining to positive regulation of cell motility and migration.

## Figures and Tables

**Table 1:** Doubling times of A549 and H358 cells with various oxygen concentrations calculated from growth curve data using the Korzynska and Zychowicz formula (*n* = 3).

Cell Line	O_2_ Protocol	Doubling Time (h) ± SE
A549	Normoxia	23.45 ± 0.42
	Hypoxia—1% O_2_	30.90 ± 2.31
	Hypoxia—0.1% O_2_	53.46 ± 12.71
H358	Normoxia	33.28 ± 1.54
	Hypoxia—1% O_2_	52.34 ± 5.46
	Hypoxia—0.1% O_2_	68.59 ± 5.62

**Table 2:** Significance testing to evaluate differences in growth kinetics of each cell line at different oxygen concentrations.

Cell Line	O_2_ Protocol	*p* Values at Different Time Points (h)						
		24	48	72	96	120	144	168
A549	Normoxia vs. hypoxia—1% O_2_	ns	ns	*	****	****	****	****
	Normoxia vs. hypoxia—0.1% O_2_	ns	ns	**	****	****	****	****
	Hypoxia—1% O_2_ vs. hypoxia—0.1% O_2_	ns	ns	ns	ns	*	*	**
H358	Normoxia vs. hypoxia—1% O_2_	ns	ns	**	****	****	****	****
	Normoxia vs. hypoxia—0.1% O_2_	ns	ns	****	****	****	****	****
	Hypoxia—1% O_2_ vs. hypoxia—0.1% O_2_	ns	ns	*	ns	ns	ns	ns

ns: not significant, *: *p* < 0.05, **: *p* < 0.01, ****: *p* < 0.0001; *n* = 3.

**Table 3:** Significance testing to evaluate differences in the cell cycle distribution of each cell line at different oxygen concentrations.

Cell Cycle Phase	O_2_ Protocol	Statistical Significance of Difference between Cell Lines (*p* Value) at Different Time Points (h)				
		2	6	12	18	24
G1	Normoxia	ns	ns	ns	ns	*
	Hypoxia—1% O_2_	ns	*	ns	*	**
S	Normoxia	ns	ns	ns	ns	*
	Hypoxia—1% O_2_	ns	ns	ns	ns	ns
G2	Normoxia	ns	ns	ns	ns	ns
	Hypoxia—1% O_2_	ns	ns	ns	ns	*

ns: not significant, *: *p* < 0.05, **: *p* < 0.01; *n* = 3.

## Data Availability

All data are shown in graphs and tables within this manuscript or provided in the Supplementary Materials.

## Supplementary Materials

The following supporting information can be downloaded at: https://www.mdpi.com/article/10.3390/cancers15092472/s1, File S1: Differential gene expression of hypoxic A549 and H358 cells determined by RNA sequencing.
